# Safranal induces DNA double-strand breakage and ER-stress-mediated cell death in hepatocellular carcinoma cells

**DOI:** 10.1038/s41598-018-34855-0

**Published:** 2018-11-16

**Authors:** Ala’a Al-Hrout, Amphun Chaiboonchoe, Basel Khraiwesh, Chandraprabha Murali, Badriya Baig, Raafat El-Awady, Hamadeh Tarazi, Amnah Alzahmi, David R. Nelson, Yaser E. Greish, Wafaa Ramadan, Kourosh Salehi-Ashtiani, Amr Amin

**Affiliations:** 1 Biology Department, College of Science, UAE University, P.O. Box 15551 Al-Ain, UAE; 2 Laboratory of Algal, Synthetic, and Systems Biology, Division of Science and Math, New York University Abu Dhabi, P.O. Box 129188 Abu Dhabi, UAE; 3 Center for Genomics and Systems Biology (CGSB), Division of Science, New York University Abu Dhabi, P.O. Box 129188 Abu Dhabi, UAE; 4 College of Pharmacy and Sharjah Institute for Medical Research, University of Sharjah, Sharjah, UAE; 5 Department of Chemistry, UAE University, Al-Ain, UAE; 6 Zoology Department, Cairo University, Giza, Egypt

## Abstract

Poor prognoses remain the most challenging aspect of hepatocellular carcinoma (HCC) therapy. Consequently, alternative therapeutics are essential to control HCC. This study investigated the anticancer effects of safranal against HCC using *in vitro*, *in silico*, and network analyses. Cell cycle and immunoblot analyses of key regulators of cell cycle, DNA damage repair and apoptosis demonstrated unique safranal-mediated cell cycle arrest at G2/M phase at 6 and 12 h, and at S-phase at 24 h, and a pronounced effect on DNA damage machinery. Safranal also showed pro-apoptotic effect through activation of both intrinsic and extrinsic initiator caspases; indicating ER stress-mediated apoptosis. Gene set enrichment analysis provided consistent findings where UPR is among the top terms of up-regulated genes in response to safranal treatment. Thus, proteins involved in ER stress were regulated through safranal treatment to induce UPR in HepG2 cells.

## Introduction

Despite all efforts, more people are diagnosed with hepatocellular carcinoma (HCC); the most common type of primary liver cancer and the second leading cause of cancer-related death worldwide^1^. Multiple risk factors contribute to HCC development including chronic hepatitis (B and C) infection that accounts for 70%-90% of HCC cases by providing a permissive environment for HCC development^2^. Other HCC risk factors include alcoholism, non-alcohol fatty liver disease, iron overload, and environmental carcinogens^3,4^. Early stages of HCC show no symptoms, thus most patients are diagnosed at advanced stages. In addition, HCC exhibits a high rate of recurrence after resection or ablation; and is considerably resistant to cytotoxic chemotherapy, with a very limited number of available treatments. Thus, alternative therapeutics are well justified and are desperately needed to control HCC.

Natural products have long been a part of folk medicine and have been playing an instrumental role in the development of anti-cancer drugs^5^. Thanks to their nontoxicity and low-to-non associated side effects, 40% of FDA-approved therapeutic agents are natural-based components or their derivatives^6^. Considering their great efficacy and low toxicity, natural products have been extensively studied and introduced as a chemopreventive therapy for many diseases including cancer^7^. Medicinal plants have been suggested for cancer prevention and therapy for several reasons; they contain nutritional and anti-tumor compounds, are able to delay or prevent cancer onset, can boost the physiological status and the immune system, and most importantly, they represent a great alternative and/or adjuvant option to conventional cancer treatments by alleviating or even averting their side effects^8^.

Saffron (the stigmas of the flower of *Crocus sativus*), is increasingly gaining attention as it contains many bioactive molecules with health promoting properties; including crocin, crocetin, picrocrocin, and safranal. Previous studies have reported the anti-cancer activity of saffron and its derivatives against a wide range of cancers^9–12^. While saffron’s derivatives have been reported to inhibit the growth of HeLa cells^13^, safranal has specifically been shown to exert potent anti-inflammatory, antioxidant and anti-cancer properties^14^; and was found to induce apoptosis in both alveolar human lung cancer A549^15^, and human prostate cancer PC-3 cell lines^12^. Despite all its anti-tumor activities, the mechanism through which safranal exerts its anti-cancer effect is yet to be fully understood.

In this study, we explored the molecular mechanism by which safranal imparts its anticancer activity against liver cancer *in vitro*. We investigated the effects of safranal treatment on general aspects of HepG2 cells, such as cell viability, morphology, survival, and cell cycle progression. Here, we also reported for the first time safranal’s role in promoting DNA damage through inducing DNA double-strand break (DSB) and inhibiting DNA repair mechanisms. Apoptosis was induced upon safranal treatment, which was evident from Flourescence Activated Cell Sorting (FACS) analysis data and activation of both initiator and executioner caspases. Finally, the present results provided evidence that the herein reported safranal-induced apoptosis was mediated through endoplasmic reticulum (ER)-stress.

## Results

### Safranal Inhibits Growth and Survival of HepG2 Cells

**Figure 1:**
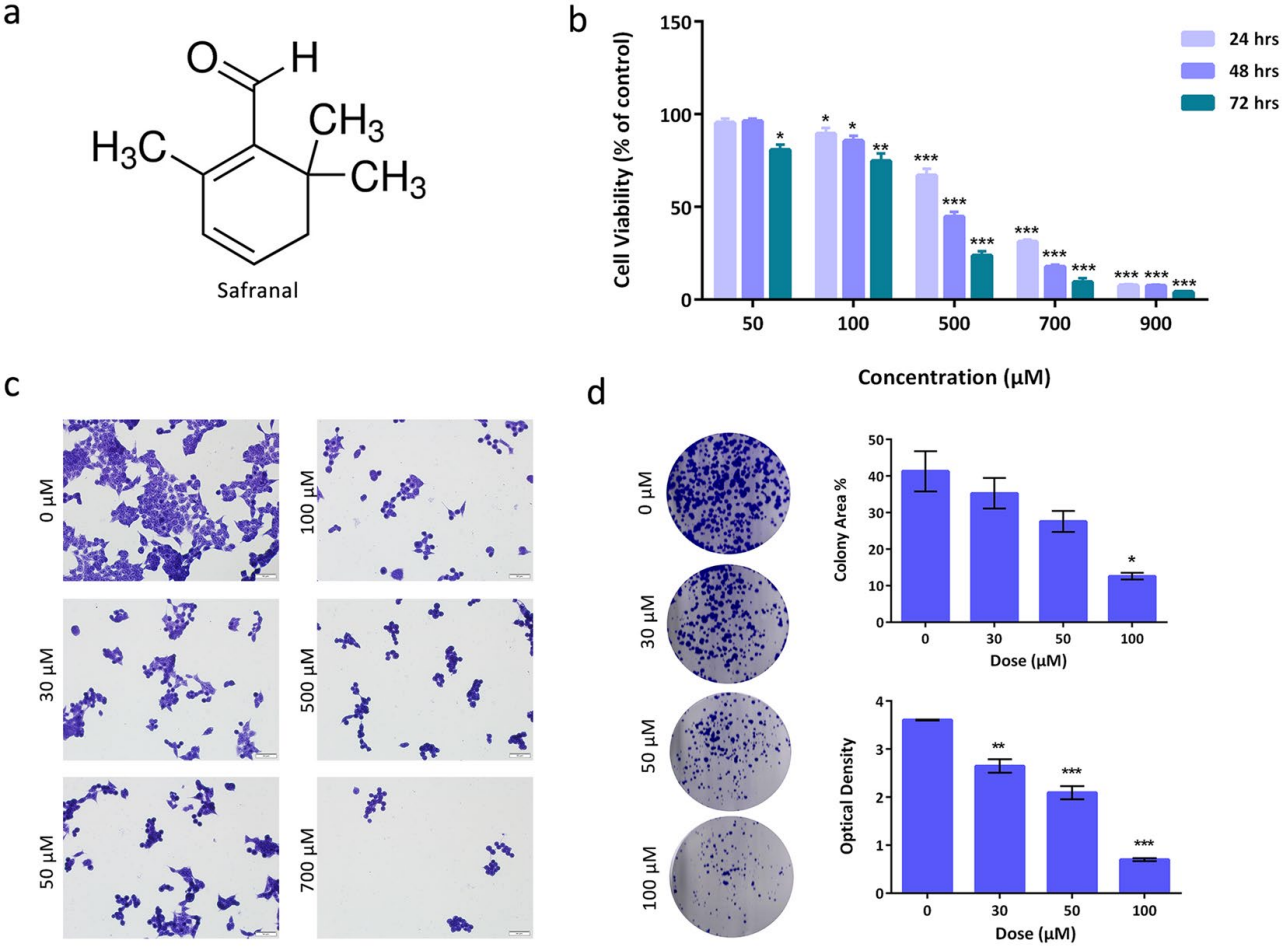
Safranal inhibits growth and survival of HepG2 cells. (**a**) Chemical structure of safranal. (**b**) Cell viability of HepG2 cells after treatment with different concentrations of safranal for 24, 48 and 72 h. (**c**) Assessment of morphological changes of safranal-treated HepG2 cells (24 h). Cells were fixed and stained with crystal violet. (**d**) Representative images of colony formation assay of HepG2 cells treated with different concentrations of safranal (24 h). The effects of safranal treatment were quantified by calculating percent of area occupied by colonies in treated and non-treated samples (representative of triplicate samples) and absorbance of each treated and non-treated wells (representative of biological triplicates, each in technical triplicate). T-test was carried out (* p ≤ 0.05, ** p ≤ 0.001, ***p ≤ 0.0001).

To assess the cytotoxic effects of safranal (Fig. 1a) on liver cancer *in vitro*, HepG2 cells were treated with a range of concentrations (50–900 μM) of safranal for 24, 48, 72 h. Treatment with safranal resulted in dose- and time-dependent inhibition of cellular viability (IC_50_ 500 μM; Fig. 1b). Cells treated with increasing doses of safranal for 24 h exhibited morphological alterations including more rounded cell shapes, cell shrinkage, and increased detachment. Safranal-induced morphological changes were particularly evident after treating cells with a dose of 500 μM (Fig. 1c). Colony formation assay was also performed to assess the effects of safranal on the survival of HepG2 cells. Cells were treated with a range of concentrations (30–100 μM; higher doses eradicated all colonies) of safranal. Safranal inhibited colony formation of HepG2 cells in a dose-dependent manner, being most effective at 100 µM dose. This inhibition was clearly reflected by the lower number of visible colonies in the treated plates in comparison to the control. The decreasing numbers of colonies was quantitatively represented in smaller occupied areas and lower optical densities (Fig. 1d).

### Safranal Arrests HepG2 Cells at G2/M and S Phase and Affects Cell Cycle Regulators

**Figure 2:**
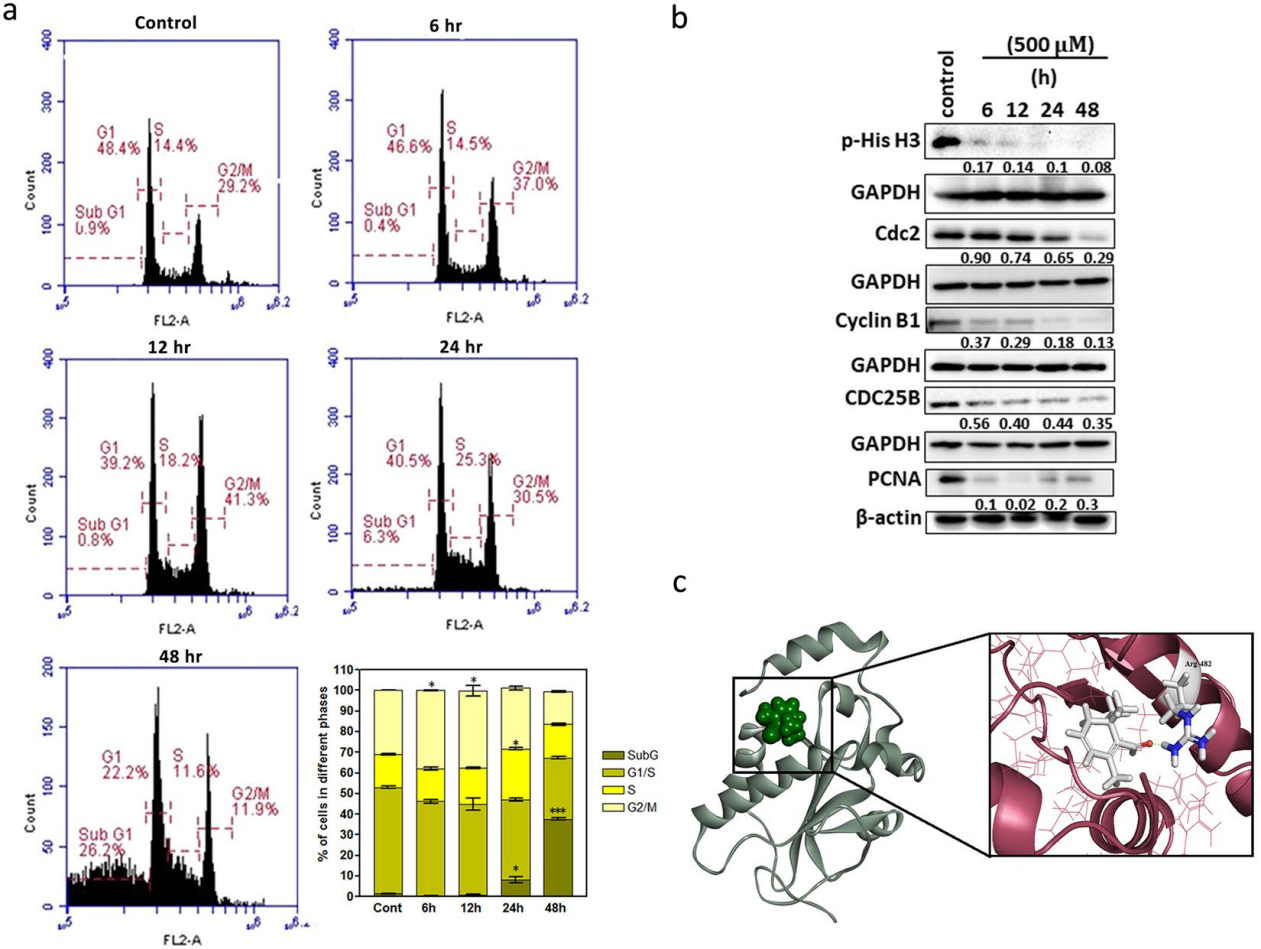
Safranal arrests HepG2 cells at G2/M and S Phase and affects cell cycle regulators. (**a**) Cell cycle progression of HepG2 cells after treatment with safranal at a dose of 500 µM over a period of 48 h; and quantitative distribution of HepG2 cells in different phases of the cell cycle at different time intervals. Statistical analysis was carried out by student’s t-test using GraphPad Prism software and p < 0.05 was considered as statistically significant. *p < 0.05 and ***p < 0.01 (**b**). Western blot analysis of cell cycle regulatory proteins in HepG2 cells post treatment with safranal at a dose of 500 µM. Each band intensity was quantified using ImageJ, normalized relative to their respective loading control bands. Values are expressed as ratio of untreated control. Western blot images (**b**) have been cropped for clarity with full blots presented in Supplementary Fig. 1. (**c**) Best docked poses of safranal within the human CDC25B binding site.

To investigate how safranal affects cell cycle progression, cell cycle distribution was analyzed by flow cytometry. Treatment with 500 μM safranal resulted in a G2/M phase arrest at 6 and 12 h post treatment, and an S-phase arrest at 24 h. Additionally, safranal induced significant (p < 0.001) increase in sub-G population post 24 and 48 h of treatment, indicating that safranal induced apoptosis of HepG2 cells (Fig. 2a). The effect of safranal on the protein expression of key cell cycle regulators was investigated where HepG2 cells were treated with 500 µM safranal for 6, 12, 24, and 48 h. Expression of phosphorylated histone H3, an indicator of cells entering mitosis, was inhibited dramatically post safranal treatment suggesting interruption of G2/M transition, which is also reflected in the inhibition of the proliferation marker PCNA (Fig. 2b). Cdc2/Cyclin B1 (also known as Cdk1/Cyclin B1 complex) is needed for G2/M transition and has been shown to require CDC25B for its activation *in vitro*^16^. Interestingly, safranal was shown here to inhibit Cdc2 expression starting at 12 h while inhibiting expression of Cyclin B1 and CDC25B starting at 6 h of treatment.

To further understand the mechanism by which safranal exerts its effects on the corresponding CDC25B, a molecular docking approach was utilized with the aim of identifying the most probable binding mode and type of interactions taking place in such complex. Interestingly, safranal showed a binding profile in which the aldehyde carbonyl group involved in strong H-bond interaction with the catalytic Arg-482 of CDC25B (Fig. 2c) suggesting a direct interaction between safranal and CDC25B.

### Safranal Exerts its Cytotoxic Effect through Modulating the DNA Repair Machinery

**Figure 3:**
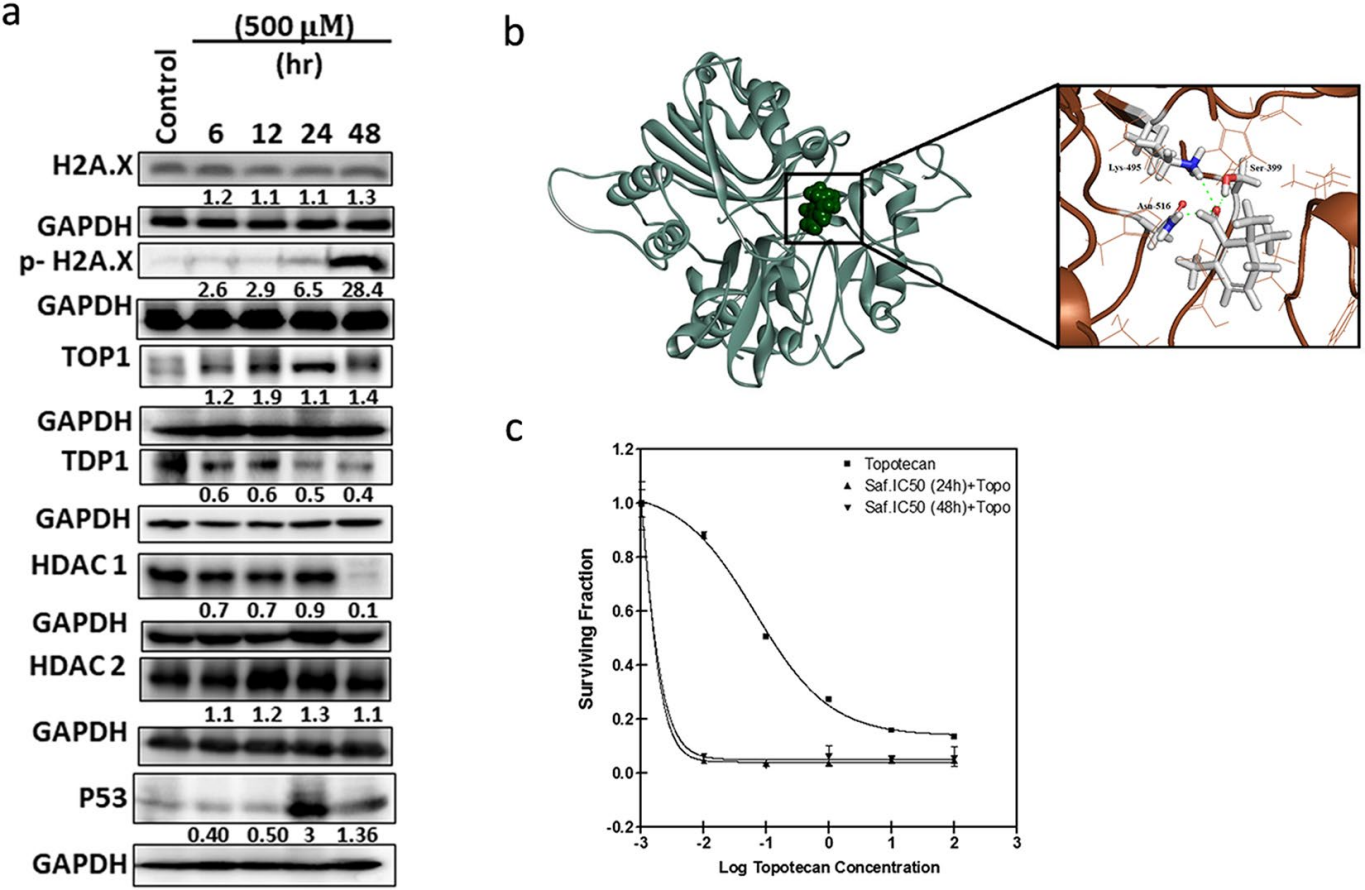
Safranal exerts its cytotoxic effect by inducing DNA damage. (**a**) Western blot analysis of key players in replication, proliferation, and DNA damage in HepG2 cells post treatment with safranal at a dose of 500 µM over a period of 48 h. Each band intensity was quantified using ImageJ, normalized relative to their respective loading control bands. Values are expressed as ratio of untreated control. Western blot images (**a**) have been cropped for clarity with full blots presented in Supplementary Fig. 2. (**b**) Best docked poses of safranal within the human TDP1 active site. (**c**) Enhancement of the cytotoxicity of topotecan by prior incubation with safranal. HepG2 cells were incubated with the topoisomerase 1 inhibitor topotecan alone or with IC50 safarnal for 24 or 48 h followed by topotecan; cell viability was measured by SRB assay.

The S-phase arrest shown by FACS analysis 24 h post safranal treatment was associated with the expression of p53, an indicator of DNA damage (Fig. 3a). Key markers of DNA replication, proliferation, and DNA damage were thus investigated to understand the effect of safranal on these processes. p-H2AX (DNA damage marker) is normally recruited to DNA break sites to form nuclear foci^17^ in cells experiencing DNA damage resulting in cell cycle arrest at G2. Interestingly, H2AX expression remained unchanged upon treatment with safranal, whereas p-H2AX was observed starting at 6 h post safranal treatment (Fig. 3a), which is in line with data reported herein of safranal-induced G2/M arrest at 6 and 12 h. Failure to repair DNA lesions has been shown to deregulate replication and transcription and lead to mutagenesis and apoptosis^18^.

Topoisomerase I (TOP1) plays a key role in DNA replication and its inhibition may lead to DNA damage which can be protected by tyrosyl-DNA phosphodiesterase (TDP1) in complex with PARP. HepG2 cells treated with safranal for 6, 12, 24, 48 h expressed higher levels of TOP1 and lower levels of TDP1, starting at 6 h (Fig. 3a). Repair of DSB is also known to be mediated by HDAC1 and HDAC2 activities. Safranal’s effect on HDAC1 expression was clear; however, the expression of HDAC2 remained unchanged. Additionally, a molecular docking experiment revealed direct interaction between safranal and the corresponding TDP1 active site (Fig. 3b). As Fig. 3c shows, pre-incubation of the cells with safranal for 24 or 48 h before topotecan greatly enhanced the cytotoxic effects of topotecan on HepG2 cells. The topotecan IC50 is reduced from 0.118 µM to 0.0016 upon incubation of the cells with safranal for 24 or 48 h before topotecan, with a sensitization factor of 73 (Table 1).

**Table 1:** IC50 of topotecan ± safranal.

Treatment	IC50 (µM)	Sensitization factor
Topotecan alone	0.118	
Safranal (24 h) + Topotecan	0.0016	73
Safranal (48 h) + Topotecan	0.0016	73

### Safranal Induced Apoptosis of HepG2 cells

**Figure 4:**
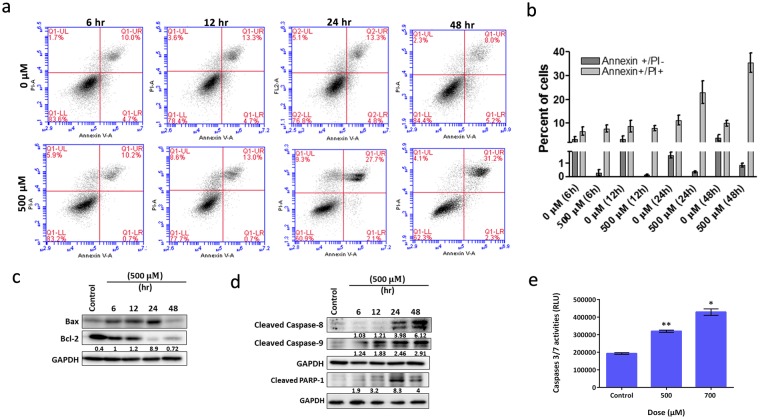
Safranal induced apoptosis of HepG2 cells. (**a**) Assessment of apoptosis by Annexin V on HepG2 cells treated with 500 µM of safranal over a period of 48 h. (**b**) Quantification of Annexin V analysis (**c**) Western blot analysis of apoptosis-related proteins in HepG2 cells treated with safranal in time-based experiments. Each band intensity was quantified using ImageJ, normalized relative to their respective loading control bands. Values are expressed as ratio of Bax to Bcl-2. (**d**) Western blot analysis of caspases in HepG2 cells treated with safranal in time-based experiments. Each band intensity was quantified using ImageJ, normalized relative to their respective loading control bands. Values are expressed as ratio of untreated control. Western blot images (**c**,**d**) have been cropped for clarity with full blots presented in Supplementary Fig. 3. (**e**) Caspase- 3/7 activity in HepG2 cells treated with 500 and 700 µM of safranal for 24 h. Student T-test was carried out (* p < 0.05, **p < 0.001, ***p < 0.0001).

Studying the effects of safranal (500 μM) on the progression of HepG2 cells through the cell cycle demonstrated a fraction of subG1 cells in the histogram indicative of apoptosis. The fraction of subG1 cells was 6.3% after 24 h and increased to 26.2% after 48 h of safranal treatment compared to 0.9% in control cells treated with DMSO (Fig. 4a). To confirm the induction of apoptosis in HepG2 cells after treatment with safranal, annexin V binding assay was employed and resulted in a significant (p < 0.01) increase in the number of dead cells from 8 to 31% after 48 h (Fig. 4a,b). To study the effect of safranal on apoptosis, changes in expressions of Bax (pro-apoptotic), Bcl-2 (anti-apoptotic), of initiator caspases (caspase-8 and -9) and of executioner caspases (caspase-3 and -7) were investigated. The ratio of Bax to Bcl-2 increased post safranal treatment in a time-dependent manner (Fig. 4c). In addition, caspase-8 was cleaved starting at 24 h, whereas caspase-9 was cleaved starting at 12 h post safranal treatment, which corresponds well with the aforementioned markers of induced DNA damage (Fig. 4d). Consistently, the activity of executioner caspases -3 and -7 increased following safranal treatment (Fig. 4e). Upregulation of pro-apoptotic proteins and the induced activity of caspases correlate well with the annexin V analysis of apoptosis.

### DEG of Safranal-Treated HepG2 Cells is Exposure-Time Dependent

To interrogate how HepG2 cells respond to treatment with safranal at the system level, cells were treated with safranal for 6, 12, 24, and 48 h, and the RNA isolated from biological triplicates were subjected to transcriptome sequencing. Following quantification of the obtained results from each sample (triplicates), differentially expressed genes (DEGs) were identified with a fold change threshold of ≥0.58 log2 value (or 1.5 fold) with FDR-adjusted p-values at 0.05. The accuracy and reproducibility of the RNAseq quantification was validated by real-time PCR (qPCR) as shown and further described in Supplementary Fig. 4.

We investigated how the safranal-treated HepG2 cells expression profiles change in comparison to the controls over time by using the short time-series expression miner (STEM) analysis algorithm The STEM clustering tool created 50 model profiles and determined which profiles had a statistically significant value by using 50 permutations per gene with standard hypothesis testing. Significant model profiles also grouped together based on similarity to form clusters of significant profiles. Of the 50 profiles, 14 showed statistically significant profiles (Supplementary Fig. 5 and Supplementary Table 1). Of those, we focused on up-and downregulated trends after safranal treatment which are represented by profiles 35, 36 (up-regulated trend) and profiles 0, 14 (downregulated trend). STEM also provides gene ontology (GO) analysis for each cluster; enriched GO terms for genes displaying downregulated trend were cell division and DSB repair (full list can be found in Supplementary Table 1). In addition, the up-regulated trend was enriched in positive regulation of protein ubiquitination and regulation of response to DNA damage stimulus.

**Figure 5:**
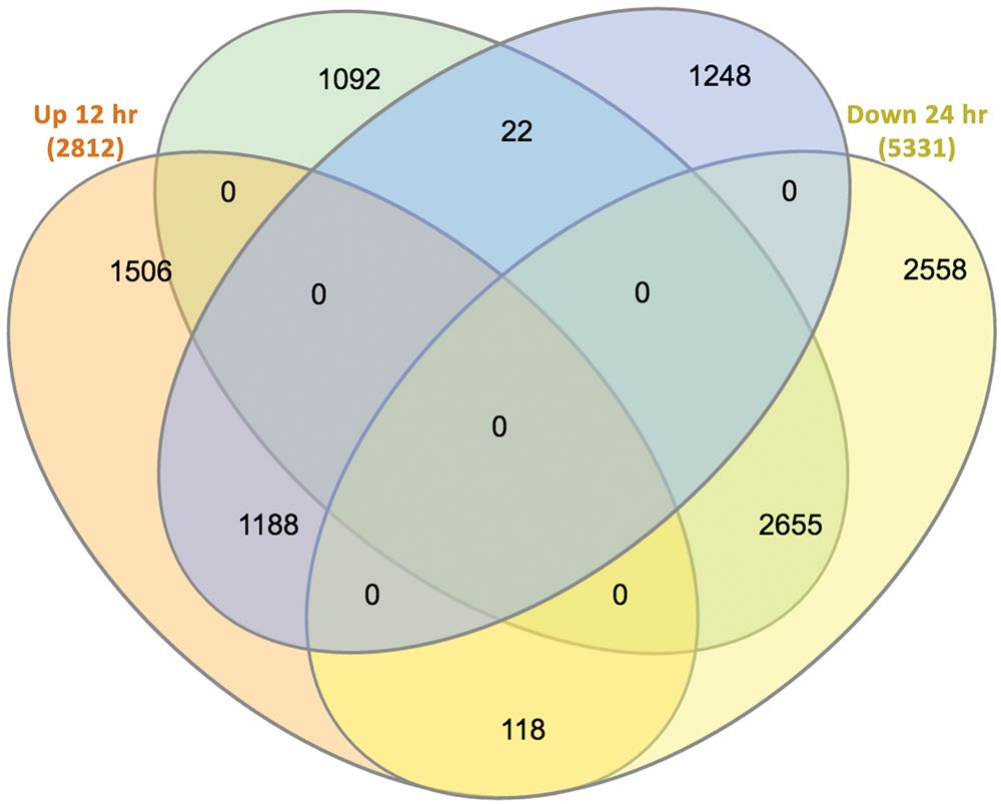
Venn diagram of differentially expressed genes at 12 and 24 h after safranal treatment. The Venn diagram shows the distribution of up and downregulated expressed genes between control and treatment after 12 h and 24 h (FDR ≤ 0.05 and fold change of ≥0.58 log2 fold (1.5 fold)). The interactive tool can be accessed online using the InteractiVenn (http://www.interactivenn.net) with Supplementary File 1.

The distribution of DEGs from safranal treatment with selected time points (12 and 24 h) was obtained relative to the control (untreated) sample. A total of 6,581 genes were significantly differentially expressed at 12 h, and 7,789 genes at 24 h. Of these time points 2,812 and 2,458 genes were upregulated respectively, and 3,769 and 5,331 were downregulated (Fig. 5, Supplementary Table 2). The numbers of DEGs uniquely appearing at 12 h posttreatment were 1,506 (upregulated) and 1,092 (downregulated), while 1,248 (upregulated) and 2,558 (downregulated) genes were uniquely appearing in the 24 h. These results suggest that the differentiation of expressed genes is time-dependent, and there are more differentially expressed transcripts when cells are treated with safranal for 24 h as compared to 12 h. We found many common genes overlapping between the two time points. In addition, there were 118 genes that were upregulated at 12 h then downregulated at 24 h; these genes were mainly involved in G1/S transition of mitotic cell cycle and cell division. Only 22 genes were, however, downregulated at 12 h then upregulated at 24 h and those were involved in proteolysis and regulation of cyclin-dependent protein serine/threonine kinase activity. These findings are collectively consistent with present immunoblot results that show safranal’s effects on cell cycle progression through inhibition of Cdc2, Cyclin B1, and CDC25B; and induction of p53.

### DEGs of Safranal-Treated HepG2 are Enriched in GO Terms Related to DNA Damage, Cell Death, and Response to Unfolded Protein

Gene ontology (GO) and gene set enrichment analyses were carried out for all DEGs with respect to biological processes using XGR software. As XGR integrates enrichment and network analyses based on input gene sets, here we focused on enrichment terms involved in cell cycle, DNA damage and other relevant pathways (Table 2, Supplementary Table 2). A number of up-regulated genes in 12 h safranal treatment were enriched in GO terms related to cellular response to DNA damage stimulus, proteasome-mediated ubiquitin-dependent protein catabolic process, and unfolded protein response (UPR), (Table 2, Supplementary Table 2). We also detected a number of downregulated genes for 12 h safranal treatment enriched in GO terms related to cell migration, growth, and wound healing. For the up-regulated genes in 24 h safranal treatment, the enriched GO terms were related to proteasome-mediated ubiquitin-dependent protein catabolic process, UPR, and apoptotic mitochondrial changes. While for the downregulated genes for the same treatment, the enriched GO terms were related to signal transduction, cell adhesion, and wound healing (Table 2, Supplementary Table 2).

**Table 2:** Summary of relevant GO enrichment for up- and downregulated genes after 12 and 24 h treatment.

Term Name	N	FDR	Term Name	N	FDR
**Upregulated 12** **h**			**Upregulated 24** **h**		
Cellular response to DNA damage stimulus	48	0.000024	proteasome-mediated ubiquitin-dependent protein catabolic process	50	9.6E-10
proteasome-mediated ubiquitin-dependent protein catabolic process	47	0.000059	Response to unfolded protein	12	0.00074
Response to unfolded protein	14	0.0008	Apoptotic mitochondrial changes	6	0.0053
**Downregulated 12** **h**			**Downregulated 24** **h**		
Cell migration	28	0.0071	Signal transduction	327	0.00011
Growth	27	0.026	Cell adhesion	149	0.00083
Wound healing	25	0.0031	Wound healing	32	0.0037

We then used the manually-curated, knowledge-based Ingenuity Pathway Analysis (IPA) designations to introduce functional relevance to up- and downregulated genes after safranal treatment for 12 and 24 h. Among the IPA generated top enriched networks were liver hyperplasia/hyper-proliferation, hepatocellular carcinoma, liver proliferation, liver necrosis/cell death and liver regeneration. The resulting networks indicated the inhibition of “hepatocellular carcinoma” at both 12 and 24 h after safranal treatments (Supplementary Fig. 6).

### Safranal Induces ER Stress in HepG2 Cells through Upregulation of Unfolded Protein Response

**Figure 6:**
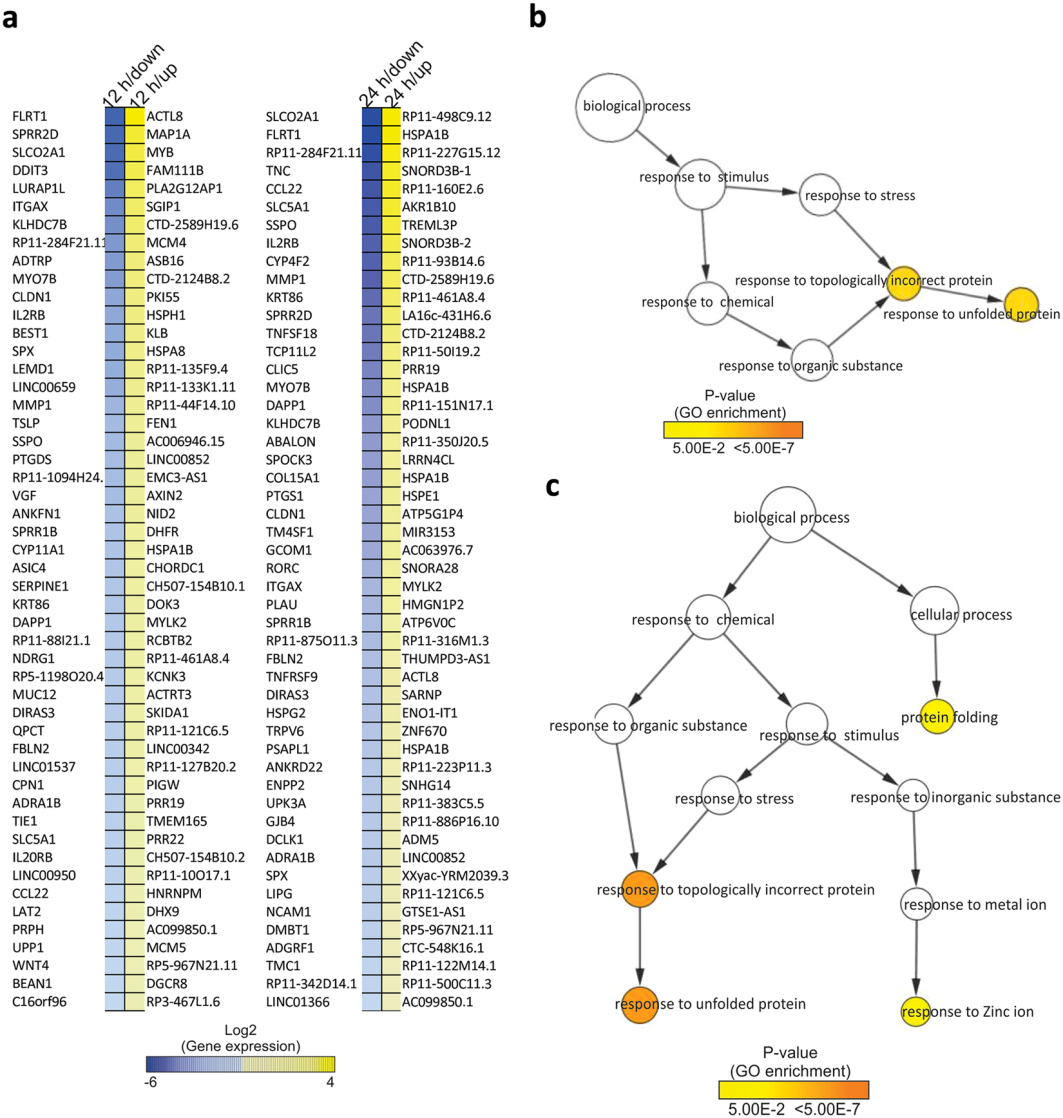
(**a**) Heatmaps of the top 50 differentially expressed genes. The heatmaps display the log2 fold change of the top 50 genes (up and downregulated) at 12 and 24 h after treatment. (**b**) GO term overrepresentation of the top up-regulated 100 genes at 12 h. (**c**) GO term overrepresentation of the top up-regulated 100 genes at 24 h. The size of each circle is correlated to the number of genes and the color of the nodes indicates different levels of significance for the enriched terms according to the provided key.

To further explore the functions associated with differentially regulated genes, we identified the top 50 up- and downregulated genes at both 12 and 24 h time points, which are displayed in a heatmap (Fig. 6a). In addition, we identified the top 100 up and downregulated genes at both 12 and 24 h time points. To carry out gene set enrichment and KEGG pathway analysis, we use BiNGO and XGR to identify the enrichment terms (Supplementary Table 2). Results from the GO network show that majority of the up-regulated genes in safranal-treated HepG2 for 12 and 24 h are involved with UPR (Fig. 6b,c).

**Figure 7:**
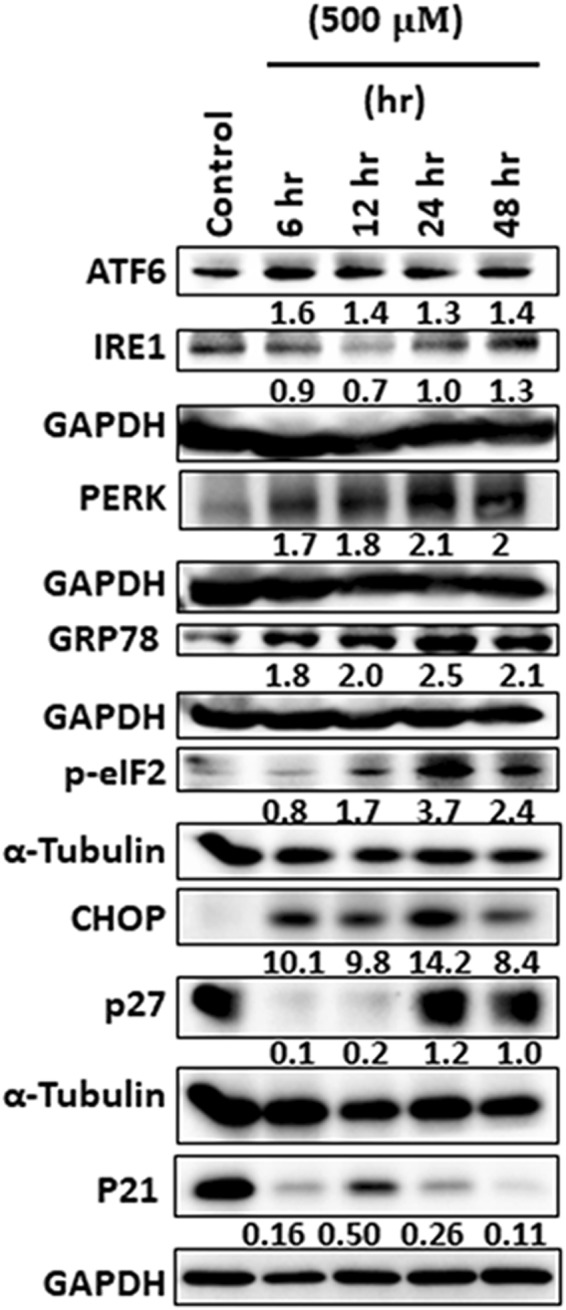
Safranal induces ER stress. Western blot analysis of key players in UPR in HepG2 cells post treatment with safranal at a dose of 500 µM over a period of 48 h. Each band intensity was quantified using ImageJ, normalized relative to their respective loading control bands. Values are expressed as ratio of untreated control. Western blot images have been cropped for clarity with full blots presented in Supplementary Fig. 7.

Assessment of ER regulators was carried out to confirm if HepG2 cells were experiencing ER stress and UPR upon treatment with safranal at different time points. The main sensors of UPR, PERK, IRE1, and ATF6 exhibited a general upregulation trend. Downstream CHOP/DDIT3 and phosphorylated eIF2α were also upregulated post safranal treatment in a time-dependent manner. Moreover, expressions of GRP78, the master UPR regulator, and of p27 were induced post safranal treatment; whereas the expression of p21 was inhibited post safranal treatment (Fig. 7).

## Discussion

Saffron and its derivatives have long been known for their capacity to impede both cancer initiation and promotion as well as promoting cancer therapy. They have also been shown to possess antitumorigenic and proapoptotic activities *in vitro*. In the present study, safranal significantly inhibited proliferation of HepG2 at 500 µM. In other studies, safranal has shown potent inhibitory effect at lower doses^11,12,19^ suggesting that HepG2 cells might be more resistant to safranal. Nonetheless, effects of safranal on HepG2 are consistent with those reported for saffron and its derivatives^20^. Many studies have reported the selective toxicity of saffron extract and its derivatives against cancer cells and its non-existent toxicity against normal cells^21^.

The ability to form colonies is essential for cancer cells survival and proliferation, where several studies have reported the ability of pro-apoptotic natural products to inhibit colony formation in different cancers^22–24^. Here too, safranal reduced the colony-forming ability of HepG2 cells in a dose-dependent manner.

Dysregulation of components of the cell cycle machinery is the common denominator of human cancers. Cancer cells often evade cell cycle checkpoints to avoid cell cycle arrest and/or apoptosis. Progression from G2 to M phase requires the formation of Cdc2 and Cyclin B1 complex, through the activity of CDC25B^16^. Indeed, inhibiting CDC25B impaired checkpoint recovery and arrested the cell cycle at the G2 phase^25^. In line with those studies and consistent with the aforementioned safranal-induced cell cycle arrest and drop in p-histone H3 level, safranal dramatically inhibited the expression of Cyclin B1 and CDC25B protein expression. Interestingly, *in silico* docking analyses revealed an interaction between safranal and the catalytic Arg-482 of CDC25B (Fig. 2c), suggesting that G2/M phase arrest of safranal-treated HepG2 cells might have been due to disruption of protein-protein interaction between CDC25B and Cdc2/Cyclin B1 complex. Lund *et al*.^26^ demonstrated inhibition of CDC25B by 2-fluoro-4-hydroxybenzonitrile through binding to a pocket in the vicinity of a protein-protein interaction hot-spot, rather than CDC25B catalytic site^26^. This is particularly intriguing as discovering or designing *de novo* inhibitors of CDC25B is quite challenging due to its shallow active site pocket^27^. However, a number of natural and synthetic compounds that show selective inhibition of CDC25B have shown promising anticancer effects in several cancers^28^. Some of those compounds displayed inhibitory effects against parental cancer cell line and their multidrug-resistant derivatives^29,30^. Other inhibitors were reported to block cell cycle progression of different cancer cells; and interestingly, some were able to inhibit cell cycle progression at both G1 and G2/M phases^28^. In agreement with those findings, safranal did inhibit cell cycle progression, through arresting HepG2 cells at both S and G2/M phases. Similar findings have been reported where UCN-01, a protein kinase inhibitor, inhibited proliferation of hepatoma cell lines including HepG2 through arresting the cell cycle at S and G2/M phase^31^.

Safranal treatment induced phosphorylation of histone H2AX that is a marker of DSB, also induced by replication stalling^32^. The elevation of p-H2AX coincided with a drop in TDP1 level suggesting that DNA breaks may result from lack of repair by TDP1. To understand how safranal induces DNA damage, we investigated a key regulator of DNA replication (TOP1) and other contributors to DNA damage repair (TDP1, PAPR, HDAC1 and HDAC2). TOP1 facilitates DNA replication by relieving supercoiling and tension of DNA via cleaving and rejoining one strand of the DNA duplex. Thus, TDP1, through forming a multiprotein complex that includes PARP^33^, is normally needed to remove TOP1–DNA cleavage complexes, thus protects against DNA strand breaks arising as a result of TOP1 malfunction. Cancer cell survival relies on accurate DNA repair, which provides an opportunity to treat tumors by DNA damaging agents. Cleaving PARP results in impairing DNA repair and accumulation of DNA damage. Similarly, as a key component in the DNA repair machinery, TDP1 inhibition can accentuate the effects of DNA damaging agents and ultimately apoptosis. This is particularly critical when developing novel therapeutic agents against cancer. DNA damage arising from conventional cancer therapy (e.g. chemotherapy and radiation) is recognized by DNA repair machinery of cancer cells which leads to drug resistance^34^. By inhibiting TDP1 and hindering DNA repair, more effective cancer therapeutics can be developed^35^. TDP1 inhibitors are scarce and only few are effective at inhibiting TDP1 expression at micromolar concentrations^36^. Here, 500 μM of safranal inhibited TDP1 expression starting at 6 h; despite the increase in the expression of TOP1. The present *in silico* docking analysis revealed an interaction between safranal and the TDP1 active site. The human TDP1 consists of two domains, namely; the N-terminal domain (residues 162–350) and C-terminal domain (residues 351–608). The active site is located between these two domains and consisted from the catalytic residues (His-263, Lys-265, His-493, Lys-495 and Asn-516). Safranal showed strong interaction pattern within the TDP1 active site where it interacted with key resides such as; Lys-495, Asn-516 and Ser-399 located at the C-terminal (Fig. 3b) suggesting an inhibitory role of safranal on TDP1 protein expression. In addition, SRB assay revealed an increased sensitivity of safranal-treated HepG2 cells to topotecan, which may indicate that pre-incubation with safranal inhibited TDP1 that is needed for the repair of topotecan-induced TOP1-DNA adducts (Fig. 3c). HDAC1 and HDAC2 participate in the DNA damage response, where they facilitate repair of DSB^37^. Indeed, cells that were HDAC1 and HDAC2 depleted have been shown to be hypersensitive to DNA-damaging agents, suggesting a defective DSB repair^37^. Safranal inhibited the expression of only HDAC1, whereas HDAC2 expression remained unchanged.

Unresolved DNA damage arising from DNA replication may trigger apoptosis^38^. When a progressing replication fork encounters unrepaired DNA damage such as single- or double-strand breaks, this leads to replication fork arrest, which may collapse the replication fork and favor cell death via apoptosis. In the present study, safranal-induced apoptosis was clearly demonstrated by the detection of subG1 cells in the cell cycle distribution, the binding pattern to annexin V, and the increased Bax/Bcl-2 ratio. Mammalian caspases are divided into initiator (caspase- 8 and 9) and executioner (caspase- 3, 6, 7) caspases; where the former activate the latter that leads to the proteolysis of key structural proteins and then to apoptosis (intrinsic and/or extrinsic pathways)^39^.

We explored if the intrinsic apoptosis pathway, frequently mediated by DNA damage, was activated upon safranal treatment. Indeed, safranal induced cleavage of caspase-9, the initiator of the intrinsic pathway, in a time-dependent manner. Interestingly, safranal also induced cleavage of caspase-8, the initiator of the extrinsic pathway, in a similar manner to caspase-9. Other natural products and derivatives have shown similar pro-apoptotic activates by activating both pathways^40–42^. Activation of both caspases 8 and 9, has been involved in apoptotic pathway activation by endoplasmic reticulum (ER) stress^43,44^; a process that safranal modulates and will be discussed later. As expected, safranal-induced activation of the initiator caspases-8 and 9 resulted in the activation of executioner caspases- 3/7 and ultimately led into induction of apoptosis in HepG2 cells.

To gain a significant insight into the mechanism of safranal’s anticancer effects against HepG2 cells, we utilized a systems biology approach to analyze how safranal functions not only on the gene/protein level, but also on pathways and network levels. To further understand how safranal affects gene expression of HepG2 cells over time, we explored how the treatment profiles change in comparison to the untreated control over time using STEM clustering algorithm. Out of 50 model profiles created by STEM algorithm, 14 profiles showed statistically significant values, profiles 0 and 4, exhibiting a downregulation trend, were enriched in GO terms related to cell division and DSB repair. This is consistent with immunoblot data showing inhibition of PCNA, TDP1, HDAC -1 and 2; and cleavage of PARP. On the other hand, profiles 35 and 36, exhibiting an upregulation trend, were enriched in GO terms related to positive regulation of protein ubiquitination, and regulation of DNA damage response (Supplementary Fig. 5; the full list of GO terms of each model profile is provided in Supplementary Table 1). Ubiquitin and its related gene products carry out their functions through covalent attachment to cellular proteins, thereby changing the stability, localization, or activity of the target protein^45^. The identified up-regulated genes encoding ubiquitin-conjugating enzymes included UBE2A, UBE2B, UBE2D1 and F-box protein 7 (FBXO7). Those enzymes mediate the ubiquitination of the proteins involved in cell cycle and lead to proteasomal degradation of target proteins.

We then focused on enriched terms involved in cell cycle, DNA damage and other relevant pathways (Table 2). Several up-regulated genes at both 12 and 24 h, were enriched in GO terms related to UPR while up-regulated genes after 12 hours of safranal treatment were enriched in GO terms related to cellular response to DNA damage stimulus; which correlates well with the findings reported herein showing an increase in DNA damage markers post safranal treatment. Down-regulated genes after 12 h of safranal treatment were, however, enriched in GO terms related to growth, wound healing and cell migration. Indeed, by inhibiting cell growth, cell migration, and wound healing, survival and development of safranal-treated HepG2 cells can be impaired. A similar pattern was demonstrated after 24 h of safranal treatment. In addition, pathway analyses revealed the regulatory networks associated with the list of differentially expressed genes (DEGs) after 12 and 24 h of safranal treatment. HCC was highlighted as one regulatory network among the top networks that fit with our set of DEGs at 12 and 24 h (Supplementary Fig. 6). More than 200 genes were associated with the HCC network. We focused on a group of genes that are associated with of DNA damage repair, cell cycle progression, proliferation, apoptosis, ER stress, growth and invasion. The resulting networks predicted the inhibition of HCC at both 12 and 24 h after safranal treatments through inducing DNA damage response (e.g. p21/CDKN1A) and interrupting DNA repair (e.g. MGMT), in addition to inhibiting proliferation, survival, and invasion (e.g. MET, TERT, MMP2, MMP9).

Gene set enrichment and KEGG pathway analysis of safranal-treated cells showed that the majority of the up-regulated genes were involved in UPR. Prolonged ER stress and UPR often lead to the accumulation of pro-apoptotic regulators, which then activate the cell death pathway^46^.

To prevent prolonged ER stress and subsequently cell death, cells restore the ER function through the activity of stress sensors, ATF6, IRE1, and PERK^47^; all of which fall under the regulation of the main ER resident chaperone GRP78/ BiP^48^. Safranal treated HepG2 exhibited an overall upregulation of ER stress sensors and induced GRP78 expression consistent with reported effects of common pharmaceutical ER stress inducers (e.g., tunicamycin and thapsigargin)^49^. Safranal also increased p27 protein levels in treated cells. P27 is upregulated under ER stress conditions to block cell cycle progression and induce growth arrest^50,51^. In contrast, safranal inhibited p21 protein levels in HepG2 treated cells. Under ER stress, p21 is suppressed which sensitizes cells to DNA damage-induced apoptosis, shifting from the pro-survival to the pro-apoptotic role of UPR^52,53^. In addition, safranal treatment upregulated expression of CHOP and phosphorylated eIF2α. CHOP is involved in ER stress-mediated apoptosis, where overexpression of CHOP results in cell cycle arrest and apoptosis^54^. Phosphorylated eIF2α is also involved in ER stress response, where phosphorylation of eIF2α inhibits protein synthesis upon apoptotic stimuli^55^. Pharmacological induction of ER stress has been shown to suppress p21 levels, concurrent with induction of CHOP, a major regulator of ER stress-related apoptosis. CHOP was, therefore, reported to mediate cell cycle through regulating p21/waf1 during ER stress driving cells into a pro-apoptotic program manifesting its dual function where in addition to inherently inducing apoptosis, CHOP also relieves the anti-apoptotic activity of p21^53^. Curcumin has been reported to inhibit ERAD activity and upregulate PERK, eIF2α, and CHOP; which sensitizes APL cells to UPR-induced apoptosis^56^. Similar effects have been reported in U266 and HepG2 cells, where treatment with anacardic acid resulted in ER stress-induced apoptosis, in time- and dose-dependent experiments^57^. Treatment with anacardic acid increased expression of ATF4, p-eIF2α, GRP78, and CHOP, suggesting that ATF4 is one of the key pathways promoting anacardic acid mediated ER stress. These data are consistent with observations made in our study, where p-GRP78 and CHOP protein levels increased post safranal treatment, in addition to activation of upstream pathways (PERK/ p-eIF2α) that promote translation of ATF4^58^; suggesting that safranal-induced ER stress could also be partially mediated through ATF4 pathway or by inhibiting the ER function in general. Persistent ER stress has been shown to activate caspase-8 which in turn activates caspase-9 and mediate apoptosis^59,60^. Biological and pharmacological ER stressors have been shown to activate caspase-8^61^. ER stress inducers can be utilized in therapeutic approaches^62^ and some are already being used clinically or undergoing preclinical assessment^63–65^.

**Figure 8:**
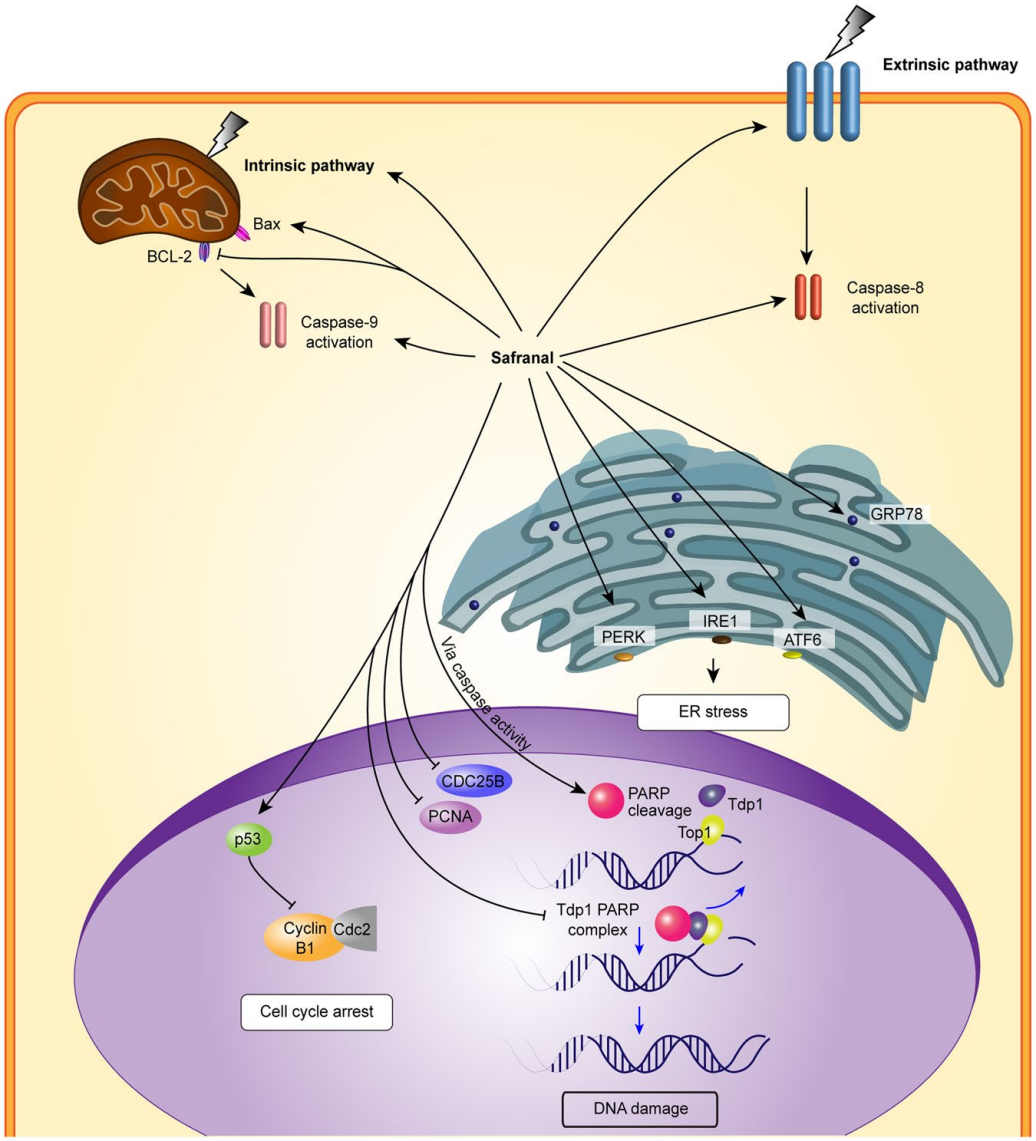
Schematic representation of safranal-mediated mechanisms against liver cancer *cells*.

In conclusion, the present study provides evidence that safranal exerts its anticancer effect in HepG2 cells by inhibiting DNA repair, resulting in increased DNA damage. This notion is evident in safranal inhibition of TDP1, a strong contributor to the DNA DSB repair mechanism, as revealed by molecular docking, immunoblotting, and SRB assay. Safranal also induced cell cycle arrest, which is reflected in inhibition of histone-H3 phosphorylation, downregulation of Cyclin B1 and Cdc2. Prolonged safranal-induced ER stress may explain the activation of both initiator caspases (caspase- 8 and -9), which leads to activation of executioner caspase-3 and -7, PARP cleavage and apoptosis. These findings were consistent with systems analysis where UPR is among the top GO terms of up-regulated genes in response to safranal treatment for 12 and 24 h. Taken together, results reported herein suggest a novel mechanism of antiproliferative activity of safranal against HepG2 liver cancer cells that relies on ER stress and UPR activation (depicted in Fig. 8).

## Methods

### Cell culture

Cells of liver cancer cell line, HepG2, were cultured in RPMI 1640 medium (HyClone) supplemented with 10% fetal bovine serum (Sigma Aldrich) and containing 1% of 100 U/ml penicillin and 100 µg/ml streptomycin (Sigma Aldrich) at 37 °C in a humidified 5% CO_2_ atmosphere. Cells were sub-cultured each 3–5 days using trypsin 0.25% (Hyclone).

### MTT assay

HepG2 cells were seeded at a density of 5000 cells/well in 96-well plates in 100 µL of complete growth medium. Cells were allowed to attach before being treated with different concentrations of safranal (Sigma Aldrich) (50 µM, 100 µM, 500 µM, 700 µM and 900 µM) for 24, 48 and 72 h. After which, the cells were treated with 3-[4,5-dimethylthiazol-2-yl]-2,5-diphenyltratrazolium bromide (MTT) (Sigma Aldrich) and incubated for 3 h. The formed formazan crystals were dissolved using DMSO and the absorbance of the resulting product was measured at 570 nm using Epoch microplate spectrophotometer (Bio-Tek). Cell viability is presented as percentile of the untreated control which was calculated accordingly: Percent of viable cells = (Abs. of treated cells/Abs. of control cells) × 100. *p ≤ 0.05, **p ≤ 0.001, ***p ≤ 0.0001.

### Cell morphology

HepG2 were seeded at a density of 0.25 × 10^6^ cells/well in 6-well plate. After allowing the cell to attached, HepG2 cells were treated without or with different concentrations of safranal (30, 50,100, 500, 700 µM) for 24 hrs. After which, cells were fixed and stained with crystal violet. The morphology of the cells was assessed after being fixed and stained with 0.5% crystal violet using IX53 microscope (Olympus).

### Colony formation

HepG2 cell were seeded at a density of 1000 cells/6-well plate, and left to incubate for 24 h to allow attachment before being treated with different concentrations of safranal (30, 50, 100 µM) for 24 h. After which, culture media containing safranal was replaced by fresh growth media without safranal. Culture media was replenished every 3 days, until visible colonies were formed. Colonies were fixed with absolute methanol, then stained with 0.5% crystal violet. Colonies were then imaged and analyzed using ImageJ plugin ColonyArea. Results are represented as the percent of area occupied by colonies. To confirm, an absorption-based method was carried out to validate results obtained from ImageJ. Briefly, stained colonies were treated with 10% acetic acid solution to dissolve the crystal violet stain. After which, 100 μL of each triplicate sample was transferred to a 96-well plate (in triplicates), and absorbance was measured using Epoch microplate spectrophotometer (BioTek). *p ≤ 0.05, **p ≤ 0.001, ***p ≤ 0.0001

### Cell cycle analysis

HepG2 cells were seeded at density of 3 × 10^6^ cells per flask in complete growth medium and were allowed to attach overnight. After which, cells were treated with 500 µM of safranal for different time intervals (6–48 h). At the indicated time intervals, cells were collected by incubation with trypsin and washed twice with PBS. Collected cells were fixed in 70% ethanol, treated with RNase and stained with propidium iodide. Cell cycle distribution was analysed by flow cytometry in a FACS scan (Becton Dickenson, Germany).

### Western blotting

HepG2 cells were seeded at a density of 1 × 10^6^ cells/100 mm plate and allowed to attached before being treated with safranal. Cells were treated with 500 µM of safranal for different time intervals (6–48 h) for time-dependent experiments. Whole cell lysates were separated using 10–15% SDS polyacrylamide gel electrophoresis. Proteins were transferred onto PVDF membranes prior to incubation with various primary antibodies p-histone H3, Cdc2, Cyclin B1, CDC25B, p21, p53, H2AX, p-H2AX, TOP1, TDP1, Cleaved PARP1, PCNA, HDAC1, HDAC2, Cleaved Caspase- 9, Cleaved Caspase- 8, Bax, Bcl-2, GRP78, ATF6, IRE1, PERK, p-eIF2S1, p27, and CHOP. GAPDH, β-actin, and α-Tubulin were used as loading controls. See Supplementary Figs 1–3 and 6 for uncropped Western blot images. Protein bands were detected using WesternSure Chemiluminescent Substrate (LI-COR) and C-DiGit blot scanner (LI-COR).

### Caspase- 3 and 7 activities

HepG2 were seeded at a density of 5000 cells/well in a 96-well plate, and were allowed to attach. After which, cells were treated with 500 and 700 µM of safranal for 24 h. Caspase- 3 and 7 activities were detected using Caspase-Glo® 3/7 Assay kit according to manufacturer instructions (Promega). Luminescent signal was detected using GloMax Discover System (Promega).

### Molecular docking

The program Autodock Vina was employed during all the docking experiments. An X-ray crystal structures for the target macromolecules namely; CDC25B and TDP1 were obtained from the RSCB protein data bank (http://www.rcsb.org/pdb/home/home.do) under the entry codes of 1QB0 and 1NOP, respectively. Subsequently, the complexed inhibitors and water molecules were extracted from the initial X-ray structures and polar hydrogens and Gastieger charges were generated using the MGL Tools. Safranal was drawn using the software ChemDraw Ultra 8.0 (Cambridge Soft Corporation, USA) and was optimized for energy and geometry using MMFF94 force field. Initially, a grid boxes were established to cover the desired target molecule with a spacing of 1.0 Å between the grid points. Later, 20 Å^3^ CDC25B box was centered toward the coordinates of (17.302 *X*, 8.987 *Y*, 13.268 *Z*), and a 14 Å^3^ TDP1 box was centered toward the coordinates of (6.387 *X*, 53.857 *Y*, 3.796 *Z*). The exhaustiveness and the number of poses were set to 12 and 10 respectively. Finally, results visualization and the 3D-best docked poses were achieved using the PyMOL molecular viewer (Schrödinger Inc., USA).

### SRB assay

The effect of safranal on the cytotoxicity of the topoisomerase 1 inhibitor topotecan was tested using the sulforhodamine-B (SRB) assay as previously described^66^. Exponentially growing HepG2 cells were seeded in 96-well plates at cell density of 1 × 10^4^ cells per well. After overnight incubation, cells were treated with topotecan alone (0, 0.01, 0.1, 1, 10 and 100 µM) for 48 h or with safranal IC50 (500 µM) for 24 h followed by topotecan for 48 h, or with safranal IC50 (500 µM) for 48 h followed by topotecan for 48 h. At the end of the incubation period, cells were fixed with 50% trichloroacetic acid (TCA) for 1 h at 4 °C followed by washing, staining with SRB for 30 min followed by washing and solubilization of the stain with 10 mM Tris base (pH 10.5). The optical density (OD) at each well was measured spectrophotometrically at 564 nm with an ELISA microplate reader (Meter tech. S960, USA). The IC50 values were calculated using sigmoidal concentration–response curve fitting models (Graph Pad, Prizm software).

### RNAseq libraries construction and sequencing

Total RNA was isolated from three biological replicates of safranal treatments and untreated sample using RNeasy Mini Kit (Qiagen) following the manufacturer’s instructions. The RNAseq libraries were prepared using TruSeq RNA sample prep kit (Illumina, Inc.) following the manufacturer’s instructions. Briefly, TruSeq RNA sample prep kit converts the poly-A containing mRNA in total RNA into a cDNA library using poly-T oligo-attached magnetic bead selection. Following mRNA purification, the RNA is chemically fragmented prior to reverse transcription and cDNA generation. The fragmentation step results in an RNAseq library that includes inserts that range in size from approximately 100–400 bp. The average insert size in an Illumina TruSeq RNA sequencing library is approximately 200 bp. The cDNA fragments then go through an end repair process, the addition of a single ‘A’ base to the 3′ end and then ligation of the adapters. Then, the products are purified and enriched with PCR to create the final double stranded cDNA libraries. Finally, libraries quality control and quantification were performed with a Bioanalyzer Chip DNA 1000 series II (Agilent) and sequenced directly using the high-throughput Illumina HiSeq sequencing system (Illumina, Inc.).

### Alignment and analysis of Illumina reads against the reference genome

The data was processed through the standard RNAseq analysis pipeline at NYUAD. Briefly, alignments were performed using tophat2 v2.1.0 with the parameters “–no-novel-junctions” and “–G” when specifying the genome file. Following the tophat2 alignment stage, read counts were generated using HTseq count, and the counts were analyzed using the DESeq2 R library. The reference genome and GFF annotation correspond to the *Homo sapiens* GRCh38.p2 genome version. Venn diagram summarizing the gene expression analysis was constructed using the web-based tool InteractiVenn. Heatmaps were produced by excel.

### Quantitative real-time PCR (qPCR)

For qPCR, cDNA corresponding to 50 ng of total RNA was used per transcript to be quantified. Quantitative PCR reactions were performed on an Applied Biosystems StepOnePlus instrument system using KAPA SYBR FAST One-Step qRT-PCR Kit (Kapa Biosystems, USA) with gene-specific primers according to the manufacturer’s instructions. Data were normalized relative to Hprt1 and Actb gene values, which exhibited stable expression levels between safaral treatments and the control samples (Supplementary Fig. 1). Melting curves were performed on the product to verify that only a single product was amplified without primer dimers and other bands; melting curve analysis was performed for each primer pair before further analyses. Relative quantitative analysis was performed by comparative quantitation using StepOne v2.3 software. All reactions were run in triplicate. The primers for the qPCR reactions are listed in Supplementary Table 2.

### Differential gene expression trend analysis

To analyze the trend of gene expression profiling between control compare to treatment from four-time points based on FPKM values, Short Time-series Expression Miner (STEM) software (http://www.cs.cmu.edu/~jernst/stem) was used to compare the trends exhibited in safranal treatment. P-values correspond to the differential gene expression test, which was performed to analyze all trends in these four-time points. STEM determines statistically significant gene expression profiles by comparing the ratios relative to the first time point (here is control). Thus, the first value is always 0. The STEM clustering method was selected with the default parameters; STEM determines profiles statistically significantly enriched by comparing the number of genes assigned with what would be expected based on permutation with Bonferroni correction for multiple comparisons.

### Gene set enrichment analysis

Functional and gene set enrichment analysis of DEGs was performed using eXploring Genomic Relations (XGR) which is an open source tool for enrichment analysis with default parameters. The enrichment test is based on Hypergeometric distribution to identify the enriched gene ontology terms. The false positive rate was calculated by simulating a random set of genes of different sizes and found they were independent of the size of gene sets. Network analysis of over-representation GO terms was performed using the Biological Networks Gene Ontology tool (BiNGO) plug-in for Cytoscape. BiNGO retrieved the relevant GO Biological process annotation then tested for significance using the hypergeometric test and corrected multiple testing using Benjamini and Hochberg false discovery rate (FDR) correction ≤ 0.05.

### Pathway analysis

We used the Ingenuity Pathway Analysis (IPA) (QIAGEN Inc., https://www.qiagenbioinformatics.com/products/ingenuity-pathway-analysis)^67^ to examine the biological network associated with the safranal treatment at 12 and 24 h (Supplementary Fig. 6). IPA software (http://www.ingenuity.com) uses a manually curated database which contains information from several sources including published journal papers and gene annotation databases. The Fisher’s exact test was used to calculate the probabilities between input gene set with the canonical pathway, disease and tox function. IPA also predicted the upstream and downstream effects of activation or inhibition on other molecules based on the input gene set’s expression data.

## Electronic supplementary material

Supplementary information

Table 1

Table 2
