# Supplementary material for: Safranal induces DNA double-strand breakage and ER-stress-mediated cell death in hepatocellular carcinoma cells
Source: Sci Rep. 2018 Nov 16;8:16951. doi: 10.1038/s41598-018-34855-0 (PMC6240095; doi:10.1038/s41598-018-34855-0)
Supplement: Supplementary file 1 — Supplementary information [file 41598_2018_34855_MOESM1_ESM.pdf]

Supplementary Information

Safranal induces DNA double-strand breakage and ER-stress-mediated cell death in hepatocellular carcinoma cells

Ala'a Al-Hrout[1], Amphun Chaiboonchoe[2], Basel Khraiwesh[2,3], Chandraprabha Murali[1], Badriya Baig[1], Raafat El-Awady[4], Hamadeh Tarazi[4], Amnah Alzahmi[2], David R. Nelson[2], Yaser E. Greish[5], Wafaa Ramadan[4], Kourosh Salehi-Ashtiani[2, 3]†, Amr Amin[1, 6]*

[1]Biology Department, College of Science, UAE University, P.O. Box 15551, Al-Ain, UAE

[2]Laboratory of Algal, Synthetic, and Systems Biology, Division of Science and Math, New York University Abu Dhabi, P.O. Box 129188, Abu Dhabi, UAE

[3]Center for Genomics and Systems Biology (CGSB), Division of Science, New York University Abu Dhabi, P.O. Box 129188, Abu Dhabi, UAE

[4]College of Pharmacy and Sharjah Institute for Medical Research,

University of Sharjah, Sharjah, UAE

[5]Department of Chemistry, UAE University, UAE

[6]Zoology Department, Cairo University, Egypt

*Corresponding to: a.amin@uaeu.ac.ae , Biology Department, UAE University, P.O. Box 15551, Al-Ain, UAE, Tel : 97137136519 – Fax: 97137134927

†Corresponding to: ksa3@nyu.edu , Center for Genomics and Systems Biology (CGSB), Division of Science, New York University Abu Dhabi, P.O. Box 129188, Abu Dhabi, UAE

SUPPLEMENTARY FIGURES

Supplementary Figure 1. Uncropped blots of figure 2.

FIGURE 2(b)

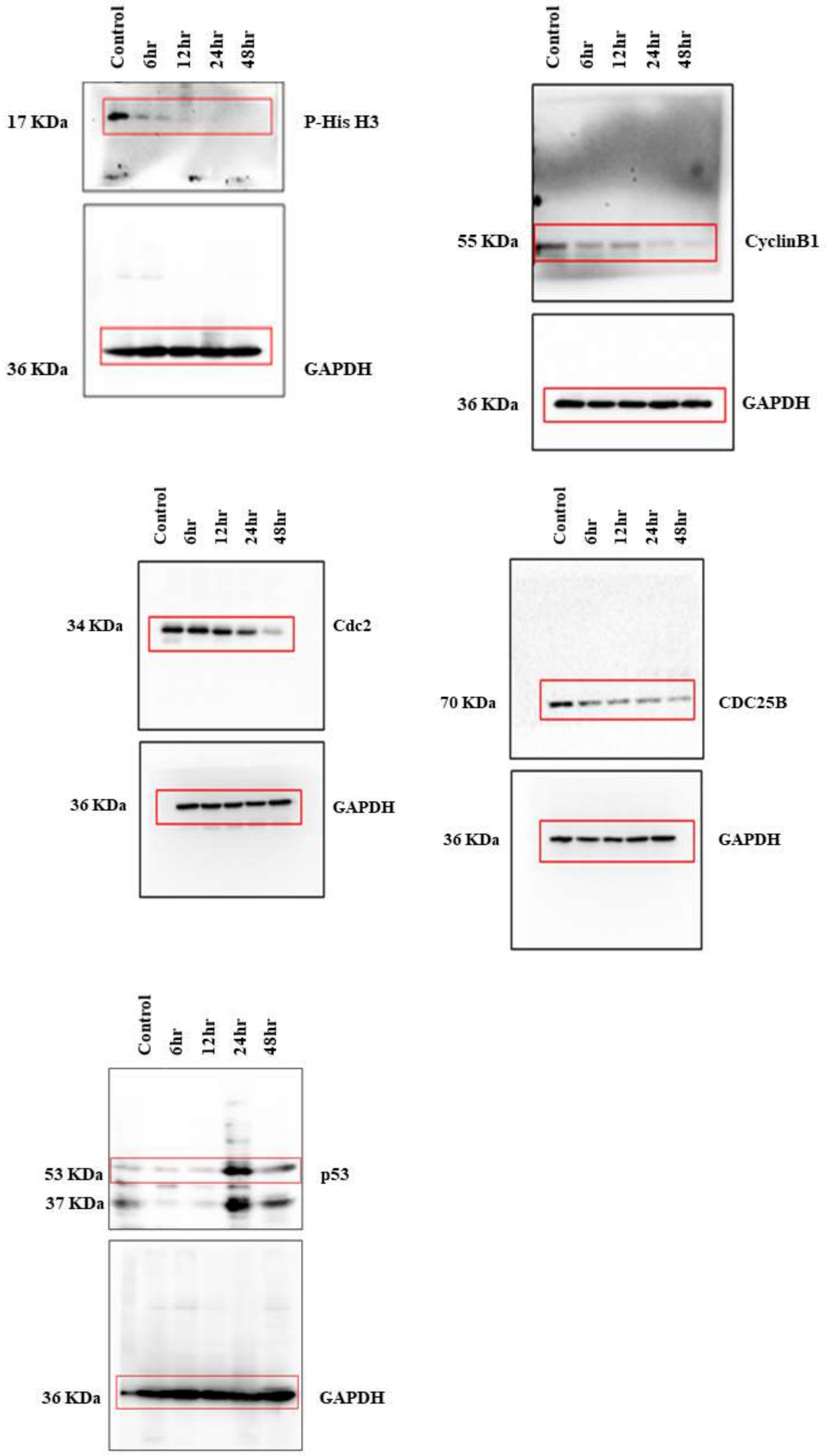

Supplementary Figure 2. Uncropped blots of figure 3.

FIGURE 3(a)

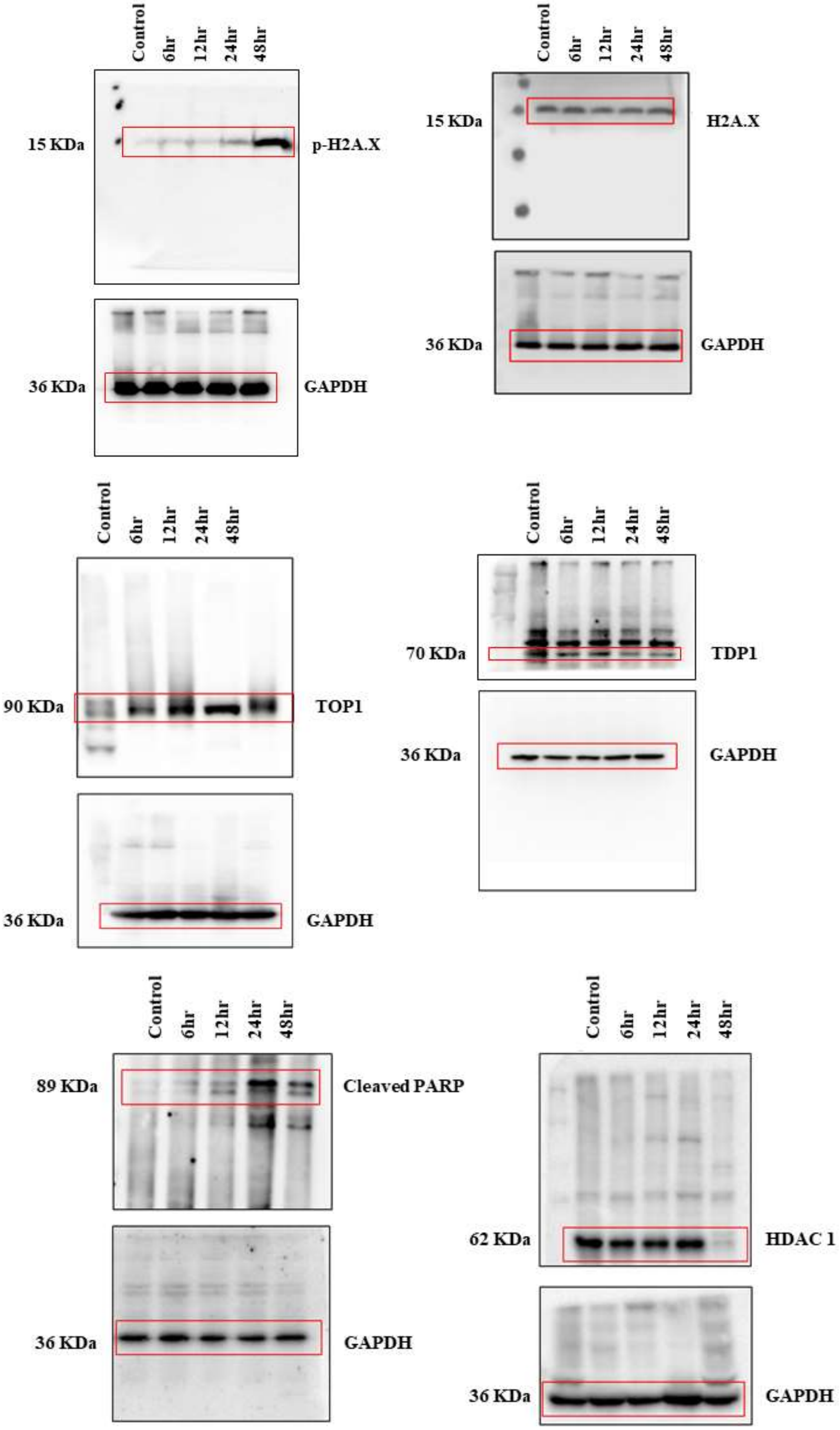

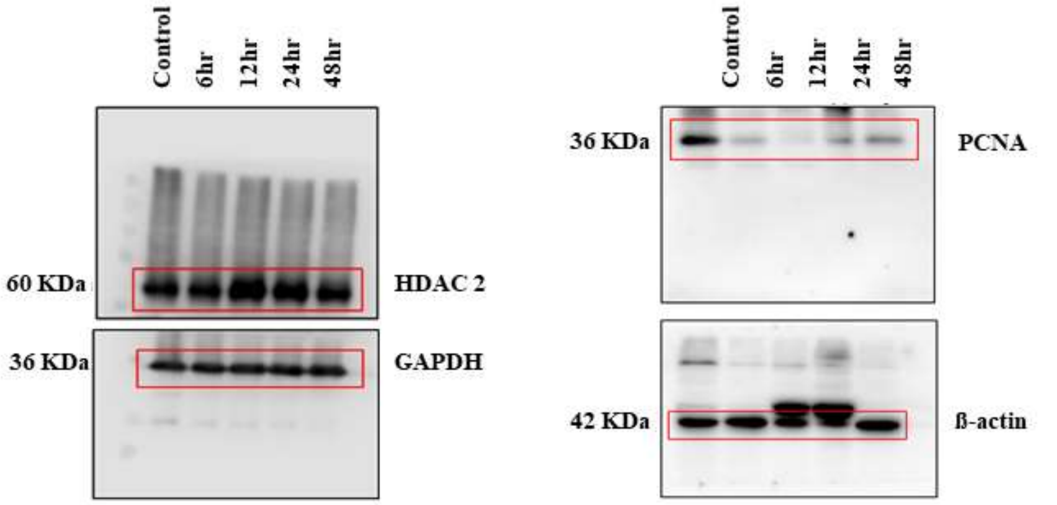

Supplementary Figure 3. Uncropped blots of figure 4.

FIGURE 4(c,d)

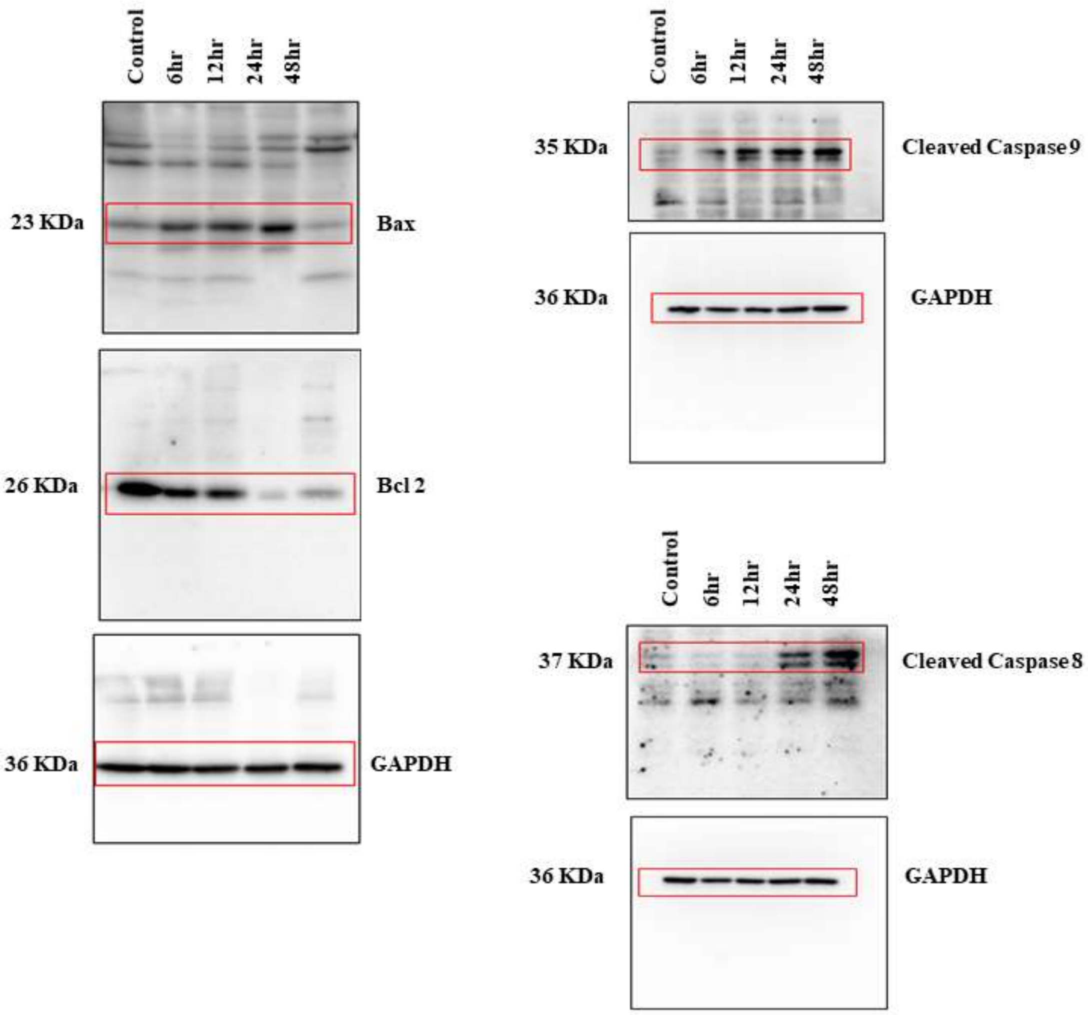

Supplementary Figure 4.

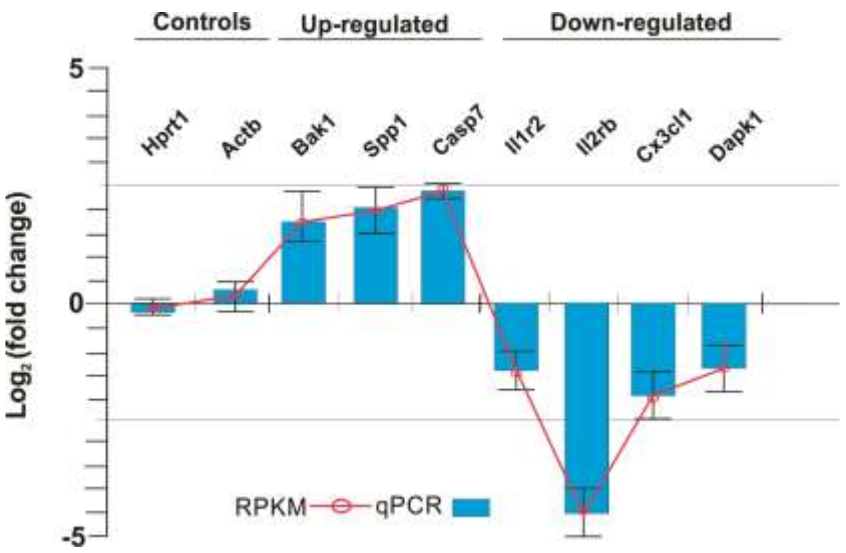

Supplementary Figure 4. Validation of RNA-Seq gene expression data. Expression profiles of a representative set of genes were validated with quantitative real-time PCR (qPCR) using RNA isolated from three biological replicates. Blue bars indicate the expression level and Log_2 (fold changes) obtained from qPCR. Red lines indicate the expression level and Log_2 (fold changes) from RNA-Seq data analyses. Error bars represent the standard error of the mean.

To validate the accuracy and reproducibility of the RNASeq quantitation, we used quantitative real-time PCR (qPCR) as an orthogonal method of gene expression analysis. The transcriptional levels of two reference genes (Hprt1 and Actb) and 8 DEGs were independently analysed by qPCR (Supplementary Figure 4). Based on the log_2 fold change, the two genes (Hprt1 and Actb) are constitutively expressed with no significant difference among safranal treatments and control sample (Supplementary Figure 4). The qPCR results also showed the same expression patterns with no difference in expression among the samples tested (Supplementary Figure 4); hence, they were considered endogenous controls (reference genes) for qPCR data normalization. The transcript levels of the 8 DEGs were obtained by the qPCR assays, normalized with the above reference genes and compared with a Log2 Ratio calculation from the RNA-Seq data. The transcript levels for the 8 selected genes were differentially regulated under safranal treatment, and the expression patterns showed high degrees of concordance between qPCR assays and RNAseq analyzed data (Supplementary Figure 4).

Supplementary Figure 5.

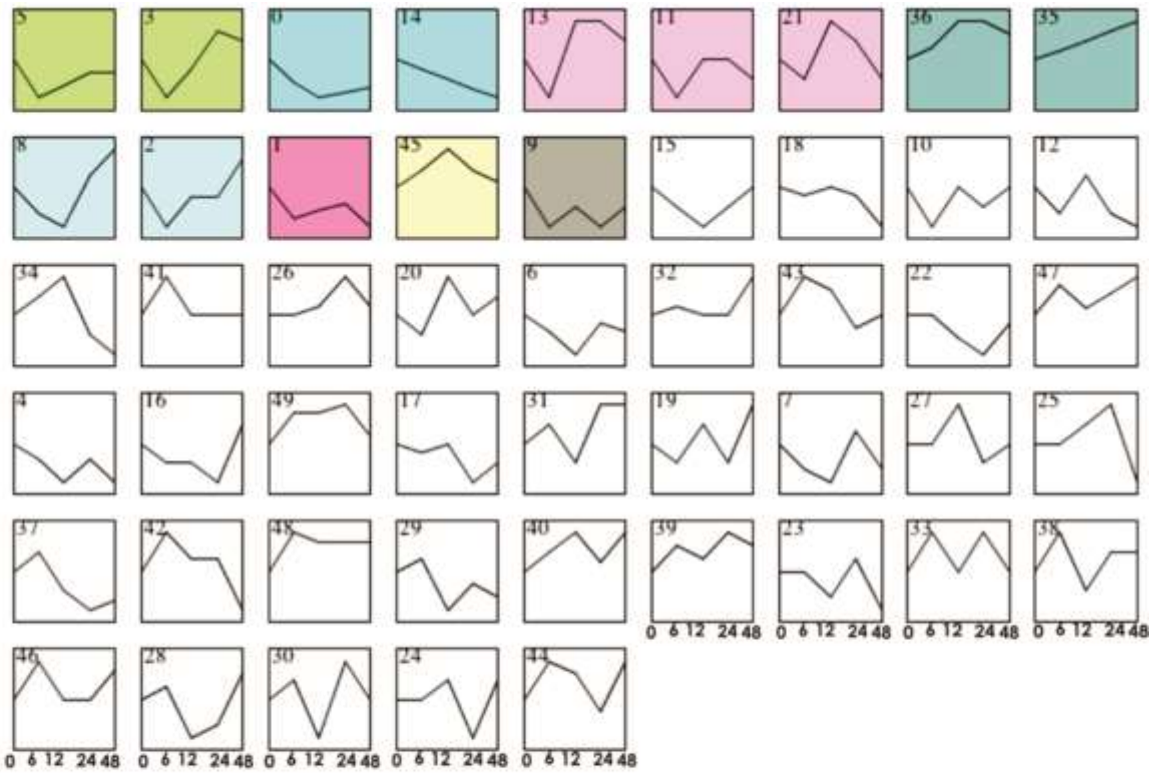

Supplementary Figure 5. Short-time series expression miner (STEM) clustering significant of the differentially expressed gene. Each box corresponds to a pre-established expression profile (number 0 to number 49) and corresponds to the changes in the expression level during time-course between control and four-time points after treatment. Clusters ordered based on number of genes and profiles ordered by significance (default). Colors indicate similar profile.

Supplementary Figure 6.

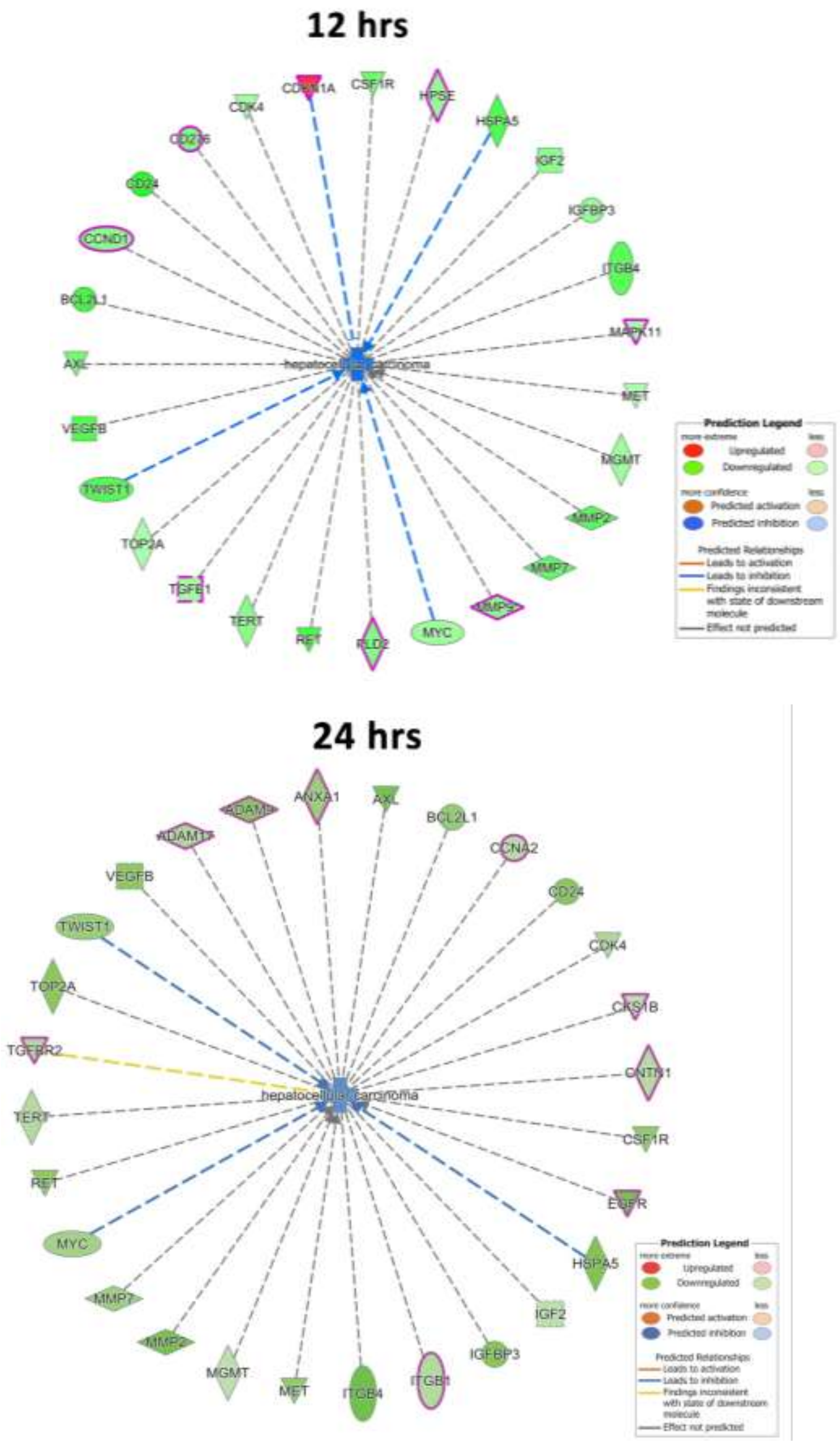

Supplementary Figure 6. The predicted inhibition of hepatocellular carcinoma by safranal treatment. Safranal treatment resulted in the inhibition of hepatocellular carcinoma at 12 h after Safranal treatment and (b) at 24 h after Safranal treatment. The inhibition was predicted based on RNA-Seq data by using IPA. Genes were colored based on their log2fold expression (Red for upregulated and green for downregulated). The color intensity is proportional to the log2fold change. The process is colored with their predicted activation stages: orange (activated) and blue (inhibited). Edges connecting the nodes are colored with orange when upstream regulators have activating effects on their target process, blue when upstream regulators inhibit the process, and yellow edges indicate inconsistencies with the prediction based on previous findings. Interested genes were highlighted in pink.

Next, to understand the functional association between expressed genes, a network was generated through the use of IPA (QIAGEN Inc., https://www.qiagenbioinformatics.com/products/ingenuity-pathway-analysis)[67]. As IPA is a manually-curated knowledge-base, these designations introduce functional relevance to up- and down-regulated genes after safranal treatment for 12 and 24 h. IPA transforms the list of genes into a set of networks, among them, the top five enriched 'Hepatotoxicity' were liver hyperplasia/hyper-proliferation, hepatocellular carcinoma, liver proliferation, liver necrosis/cell death and liver regeneration. IPA predicted the upstream and downstream effects of activation or inhibition of other molecules based on our gene expression values after treatment. These predictions are made through integrating literature reported information with observed gene expression from our RNA-Seq data. The resulting networks indicated the inhibition of hepatocellular carcinoma at both 12 and 24 h after safranal treatments (Supplementary Figure 6).

Supplementary Figure 7. Uncropped blots of figure 7.

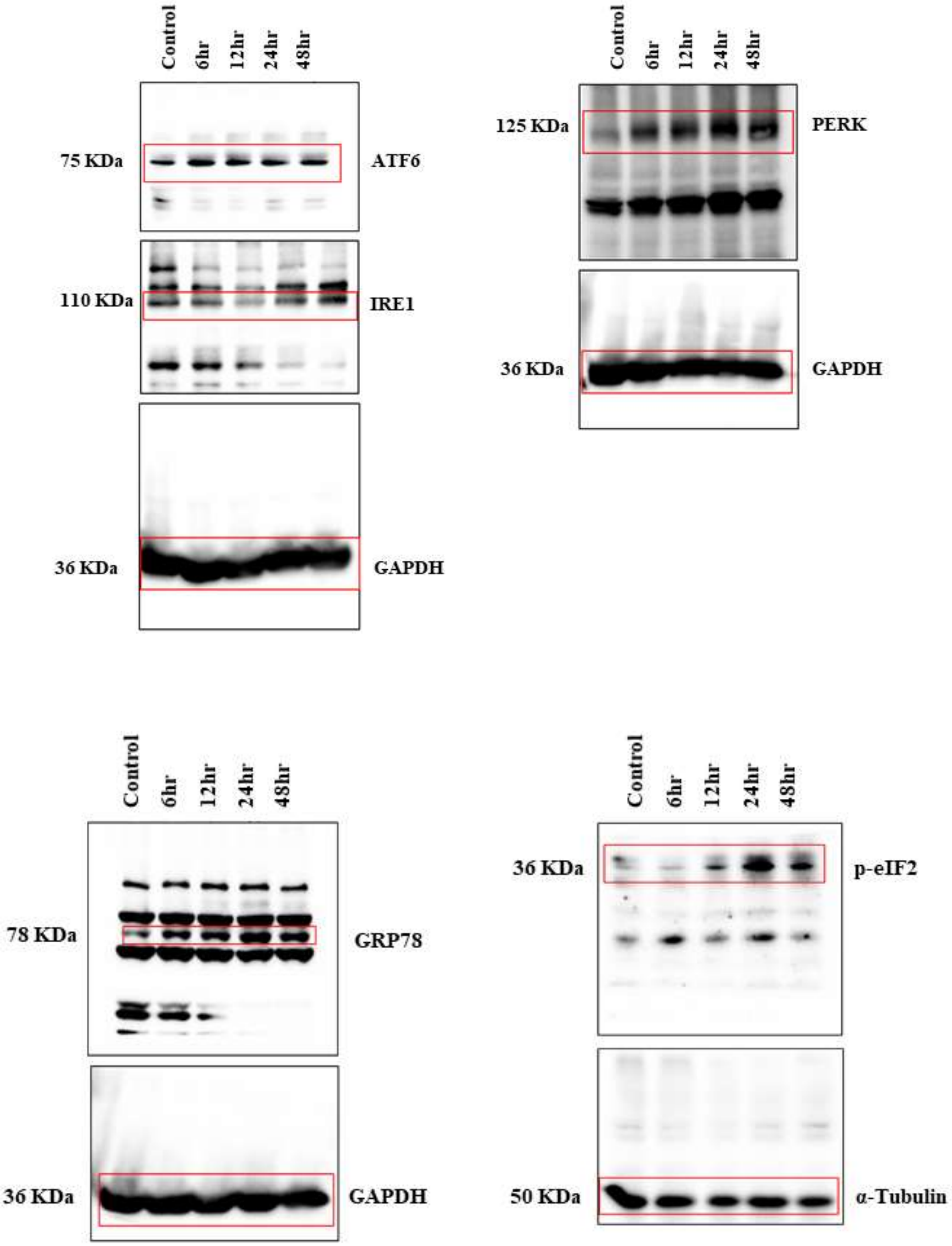

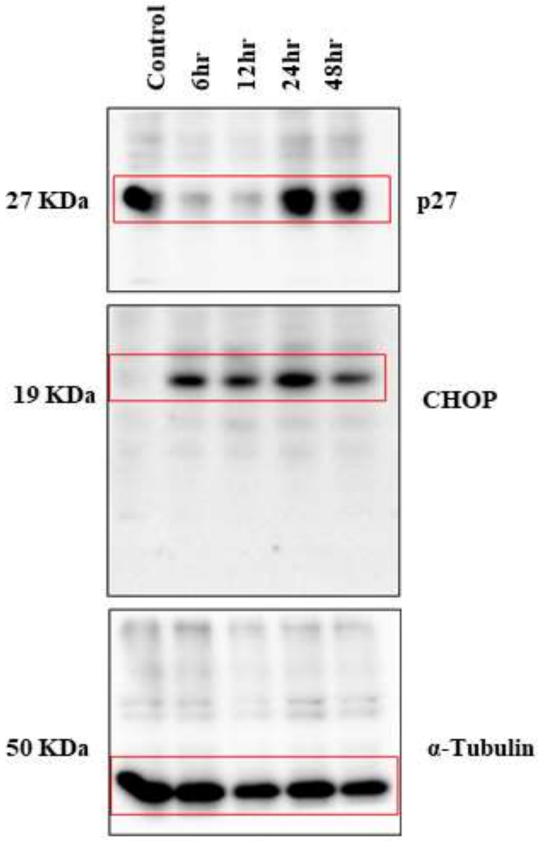
Control
6hr
12hr
24hr
48hr
27 KDa
p27
19 KDa
CHOP
50 KDa
α-Tubulin

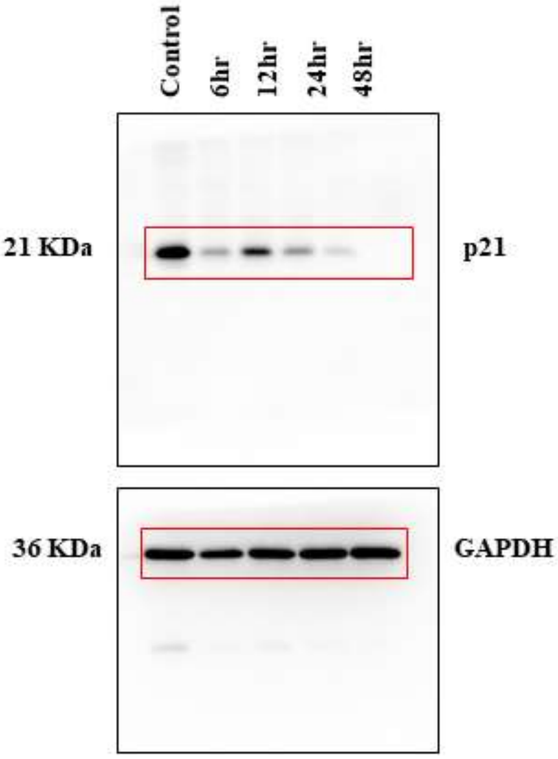
Control
6hr
12hr
24hr
48hr
21 KDa
p21
36 KDa
GAPDH
